# Validation of the Orebro musculoskeletal pain screening questionnaire in patients with chronic neck pain

**DOI:** 10.1186/s13104-018-3269-x

**Published:** 2018-03-02

**Authors:** Anke Langenfeld, Carolien Bastiaenen, Florian Brunner, Jaap Swanenburg

**Affiliations:** 1 Interdisciplinary Spinal Research ISR, Department of Chiropractic Medicine, Balgrist University Hospital, Forchstrasse 340, 8008 Zurich, Switzerland; 2 CAPHRI School for Public Health and Primary Care, Maastricht University, Maastricht, The Netherlands; 3 Department of Epidemiology, Maastricht University, Maastricht, The Netherlands; 4 Department of Physical Medicine and Rheumatology, Balgrist University Hospital, Zurich, Switzerland; 5 Physiotherapy Occupational Therapy Research Centre, Directorate of Research and Education, University Hospital Zurich, Zurich, Switzerland

**Keywords:** Validation, OMPSQ, Chronic, Neck pain

## Abstract

**Objectives:**

To validate the German version of OMPSQ (OMPSQ-G) for patients with chronic neck pain.

**Results:**

After translating OMPSQ to German, we assessed the discriminant validity between patients and healthy adults. Convergent validity was assessed using Pearson’s correlation coefficients between domains of OMPSQ-G and the German version of neck disability index (NDI-G) and visual analogue scale (VAS) of neck pain intensity. Floor and ceiling effects, internal consistency, test–retest and relative reliability were assessed. Fifty patients with chronic neck pain (mean age, 43.6 years; 34 females) and 24 healthy adults (mean age, 50.4 years; 18 females) participated. Mann–Whitney U tests showed significant differences in OMPSQ scores between both groups at the baseline (z = − 4.6; p < 0.001) and second time point (z = − 4.8; p < 0.001). OMPSQ-G scores highly and moderately correlated with NDI-G (ρ = 0.70) and VAS (ρ = 0.41) scores, respectively. There were no floor or ceiling effects. Cronbach’s alpha was 0.94. OMPSQ-G showed high reliability (intraclass correlation 2.1: 0.93; standard error of measurement, 6.9; smallest detectable change, 20 points). The Bland–Altman plot indicated no systematic error. OMPSQ-G showed good validity and reliability in patients with neck pain.

*Trial registration* NCT02540343

**Electronic supplementary material:**

The online version of this article (10.1186/s13104-018-3269-x) contains supplementary material, which is available to authorized users.

## Introduction

Neck pain is a common complaint that affects 70% of individuals at least once in their lifetime [1]. Only low back pain (LBP) causes more time off work than neck pain [2]. A frequently used assessment of LBP is the Orebro musculoskeletal pain screening questionnaire (OMPSQ), developed by Linton and Hallden [3]. OMPSQ can be used to identify patients with spinal pain [4]. OMPSQ has been translated into French, Turkish, Spanish, Chinese, Brazilian–Portuguese, Persian [5–10], including a short form of the questionnaire [11]. The short-form OMPSQ has been translated into German [12]. The original (long-form) version has not previously been translated into German and tested for its psychometric properties. To date, OMPSQ has been mainly used in patients with LBP, and few authors have reported using OMPSQ in patients with neck pain [13, 14]. They concluded that OMPSQ could be used as tool for predicting functional outcomes at 8 weeks after the initial manual therapy assessment in patients with LBP, whereas the ability for predicting outcomes of patients with neck pain is uncertain. Gabel et al. [14] used the OMPSQ to investigate patients with whiplash-associated disorder.

So far the original German version of OMPSQ has not previously been validated in patients with chronic neck pain. This study aimed to evaluate OMPSQ-G in German-speaking patients with chronic neck pain. In addition, discriminant and construct validity and reliability were evaluated.

## Main text

### Methods

#### Study design

This is a translation and validation study. At the beginning the original OMPSQ was translated and culturally adapted into German and afterwards tested for its validity and reliability in patients with chronic neck pain.

#### Questionnaire translation process

This project was authorised by the author of the original OMPSQ. Language translation was based on the original OMPSQ [3]. The cross-cultural adaptation and translation followed the guidelines for the process of cross-cultural adaption of self-report measures by Beaton et al. [15]. Two independent native German speakers translated and culturally adapted the original version. After a consensus meeting, two additional translators back-translated the German version of OMPSQ into English. The pre-final German version of OMPSQ (OMPSQ-G) was pre-tested in five healthy German-speaking volunteers, revealing no difficulties in understanding the questionnaire. Finally, the expert committee concluded that no further adaptations to OMPSQ-G were required.

#### Study sample

From November 2014 until October 2016, 50 patients with chronic neck pain were recruited from the department of chiropractic medicine at Balgrist University Hospital. All patients had chronic neck pain for at least 90 days before enrolling for the study [16] and were able to speak, read and write German. Patients were excluded for ‘red flags’ such as acute trauma, severe pain, signs of spinal cord compression and acute inflammatory arthritis.

Furthermore, a group of 26 healthy adults without neck pain were recruited from a local clinic. As recommended by the ethics committee, these subjects should not have any medical knowledge or background.

#### Study procedure

During a baseline visit, demographic characteristics were collected, and all participants were asked to independently complete three questionnaires [OMPSQ, NDI and visual analogue scale (VAS)]. After 3–7 days, all participants were asked to independently complete all questionnaires a second time at home and to send them back in a prepaid envelope. Therefore, it can be assumed that the conditions were similar when the participants filled out both the questionnaires. If the questionnaires were not returned by day 4, the participant received a reminder telephone call. Participants who did not return the questionnaires by day 7 were excluded from the study. The mean imputation was conducted for values missing from OMPSQ-G [17]. If more than three items of the questionnaire were unanswered, the questionnaire was excluded from further analyses [18].

#### Outcome measures

##### Ompsq

OMPSQ is a self-administered pain screening questionnaire that was developed to identify patients with acute or subacute musculoskeletal pain who are at risk of delayed recovery [3, 17]. A higher score indicates a higher disability. The maximum score is 210 points; a score of < 105 points indicates a low disability, that between 105 and 130 points indicates a moderate disability and that > 130 points indicates a high disability [17].

##### Ndi

NDI is a questionnaire used for assessing self-rated disability in patients with neck pain of mechanical origin [18]. It has been translated into a reliable German version (NDI-G) [19]. Scoring 0 points being the best possible score and 50 being the worst [20].

##### Vas

VAS is an reliable outcome measure used to assess pain intensity [21]. The left side of the 100 mm long line indicates “no pain,” and the end of the line on the right is “extreme pain” [22].

#### Statistical analysis

Descriptive statistics were used to describe participant characteristics. Consistency of the patients’ pain was tested using paired t-test of VAS scores.

#### Validity

To assess discriminant validity, the ability of OMPSQ-G to differentiate between healthy adults and patients with chronic neck pain was tested using Mann–Whitney U test. All other tests were conducted in the chronic neck pain sample. Criterion validity was established by correlating the total score of OMPSQ-G with those of VAS and NDI-G. Spearman’s coefficient values were interpreted as excellent (> 0.9), good (0.7–0.9), moderate (0.5–0.69), fair (0.2–0.5) or minimal-to-absent (0.0–0.2) [23]. A factor analysis with maximum likelihood extraction and varimax rotation with Kaiser normalisation was performed to assess the internal structure of the translated questionnaire. Items with loadings > 0.4 were automatically included within the matrix and items with a loading < 0.4 were inspected for clinical relevance [24]. Floor and ceiling effects of OMPSQ-G in participants were used to assess content validity. Additionally, we conducted sub-group analysis for correlations of OMPSQ-G, NDI-G and VAS in healthy participants and chronic neck pain patients.

#### Reliability

Intraclass correlation coefficients (ICCs) and their associated 95% confidence intervals (CIs) were selected to calculate the test–retest reliability of OMPSQ-G in patients with chronic neck pain [25]. ICC values of > 0.70 were considered to be acceptable [26]. In addition, internal consistency was measured. Cronbach’s α values of 0.7–0.95 were deemed to be adequate [27]. To assess absolute reliability, the standard error of measurement (SEM) and smallest detectable change (SDC) were calculated [28]. Limits of agreement (LoA) and systematic bias were assessed using Bland–Altman plots [29]. Analyses were performed using SPSS Version 22.0 statistical software (SPSS, Inc. Chicago, IL, USA), and the statistical significance level was set at 5%.

### Results

#### Translation process

The translated OMPSQ-G was pre-tested in 10 patients with complaints of chronic neck pain. The general impression of these patients was that OMPSQ-G was easy to understand. No changes were made to OMPSQ-G after the pre-test (Additional file 1).

Seventy-six participants (50 patients with chronic neck pain and 26 healthy adults) were included in this study. Two healthy adults had more than three missing items and were excluded from the analysis. Thirteen of 1050 total items (1.2%) were missing values ‘not working’ at the baseline, and 14 of 1050 items (1.3%) were missing at the second time point. Furthermore, three different items from three separate patients were missing (0.2%).

Patients’ pain levels were considered consistent because VAS scores did not significantly change (p = 0.92). Participant characteristics and results of all questionnaires at the two time points are shown in Table 1.

**Table 1:** Participant characteristics by study group

	Patients with chronic neck pain (n = 50)	Healthy adults (n = 24)
Female	34	18
Male	16	6
Age; years (SD)	43.6 (14.5)	50.5 (14.2)
Range	20–80	25–78
Weight; kg (SD)	69.9 (17.6)	67.7 (14.6)
Height; cm (SD)	169.9 (10.1)	171.5 (7.8)
Comorbidities	17	4
Employment status		
Active	43	20
Unemployed	1	0
Sick leave	0	0
Pensioner	3	3
Other	3	1
OMPSQ-G		
Baseline measurement	78.6 (25.0)	44.1 (25.2)
Second measurement	81.3 (27.8)	43.4 (23.6)
NDI-G (SD)		
Baseline measurement	13.2 (5.9)	1.6 (3.2)
Second measurement	12.5 (6.5)	1.9 (4.0)
VAS (SD)		
Baseline measurement	3.5 (2.3)	0.2 (0.1)
Second measurement	3.5 (2.4)	0.3 (0.1)

*SD* standard deviation

#### Validity

Mann–Whitney U tests showed significant differences in OMPSQ-G scores between the two groups at baseline (z = − 4.6; p < 0.001) and at the second time point (z = − 4.8; p < 0.001). All Spearman’s rho (ρ) coefficients that assessed the correlation between questionnaire scores were significant at baseline (OMPSQ-G and NDI-G at baseline, ρ = 0.71; OMPSQ-G and VAS, ρ = 0.41) and at the second time point (OMPSQ-G and NDI-G, ρ = 0.70; OMPSQ-G and VAS, ρ = 0.58). No floor or ceiling effects were observed. The lowest and highest possible OMPSQ-G scores were only found once. Sub-group analysis revealed the following correlations in healthy participants: OMPSQ-G and NDI-G (ρ = 0.355, p = 0.089, − 0.066 to 0.684 95% CI), OMPSQ-G and VAS (ρ = 0.061, p = 0.778, − 0.322 to 0.459 95% CI), NDI-G and VAS (ρ = 0.544, p = 0.006, 128–0.846 95% CI). In chronic neck pain patients the correlations were as follows: OMPSQ-G and NDI-G (ρ = 0.726, p = 0.000, 0.572–0.826 95% CI), OMPSQ-G and VAS (ρ = 0.425, p = 0.002, 0.170–0.641 95%) and NDI-G and VAS (ρ = 0.604, p = 0.000, 0.406–0.748 95% CI).

#### Reliability

**Fig. 1:**
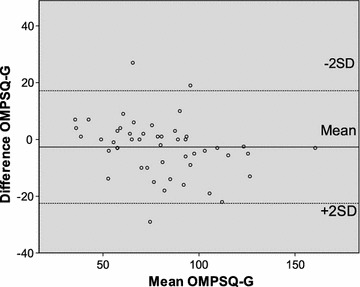
Bland–Altman plot of OMPSQ-G total scores

OMPSQ-G showed high test–retest reliability (ICC, 0.93; 95% CI, 0.88–0.96). Cronbach’s alpha was 0.94, SEM was 6.9 and SDC was 19.3 points. Bland–Altman plot (Fig. 1) indicated that all points besides three were located within the 95% LoA for test–retest reliability. No systematic error was observed.

### Discussion

This is the first translation of the long-form OMPSQ into German, which accompanies the earlier translated short-form OMPSQ [8]. As there are only a few missing values, patients and healthy participants had no problem to understand the questions, given in the questionnaire. Mean scores of OMPSQ-G were quite low in this sample of patients with chronic neck pain compared with those of a previous study in patients with acute and subacute neck pain [10]. However, the scores of OMPSQ-G were consistent with those of NDI-G and VAS; NDI-G indicated mild disability [20], and the VAS score indicated moderate pain [30]. OMPSQ-G, NDI and VAS scores showed large variance.

#### Comparison with other studies

This is the first study to show that OMPSQ-G can discriminate between patients with chronic neck pain and healthy adults. Assessment of criterion validity also revealed a good correlation between OMPSQ-G and neck disability scores and a fair correlation with pain scores and comparable with those reported in a previous study [8]. We found no floor or ceiling effects, similar to findings in the Brazilian–Portuguese version of OMPSQ [8]. Test–retest reliability (i.e. ICC) of OMPSQ-G was considered acceptable, despite the small sample size, which was considered to be acceptable given that the lower CI of ICC was above the minimum accepted level for reliability. The SDC value was smaller than that observed in the Brazilian–Portuguese version of OMPSQ-G in patients with LBP [8].

#### Clinical relevance

In conclusion, given the good validity and reliability of OMPSQ-G demonstrated in this study, the questionnaire can be considered a validated tool for identifying patients with chronic neck pain. The next research step should be to assess the predictive validity of OMPSQ-G.

### Limitations

The OMPSQ-G was not tested in patients with acute neck pain. Although a test–retest interval of 2 weeks is recommended to minimize the effect of recognition, a shorter interval was selected owing to possible varying symptoms in patients with chronic neck pain. Due to the small sample size generalising the results of this study should be performed with caution, and further analysis is recommended for future studies.

## Additional file

**Additional file 1.** Orebro musculoskeletal pain screening questionnaire in German (OMPSQ-G). Translated and validated German version of the Orebro musculoskeletal pain screening questionnaire.

## Abbreviations

CI: confidence interval
ICC: intraclass correlation coefficient
LBP: low back pain
LoA: level of agreement
NDI-G: German version of the neck disability index
OMPSQ-G: German version of the Orebro musculoskeletal pain screening questionnaire
SEM: standard error of measurement
SDC: smallest detectable change
VAS: visual analogue scale
