# Supplementary material for: Validation of the Orebro musculoskeletal pain screening questionnaire in patients with chronic neck pain
Source: BMC Res Notes. 2018 Mar 2;11:161. doi: 10.1186/s13104-018-3269-x (PMC5833147; doi:10.1186/s13104-018-3269-x)
Supplement: Supplementary file 1 — Additional file 1. Orebro musculoskeletal pain screening questionnaire in German (OMPSQ-G). Translated and validated German version of the Orebro musculoskeletal pain screening questionnaire. [file 13104_2018_3269_MOESM1_ESM.docx]

**FRAGEBOGEN zu Schulter-Nacken-Rückenschmerzen**

Name:

Adresse:

Telefon:

*Bitte lesen und beantworten Sie jede Frage sorgfältig. Überlegen Sie jedoch nicht zu lange, bevor Sie eine Frage beantworten. Es ist wichtig, dass Sie jede Frage beantworten. Es gibt immer eine zutreffende Antwort, die am ehesten auf Ihre Situation zutrifft.*

| BEISPIELE:  Bitte beantworten Sie nachfolgende Fragen, indem Sie   - eine Antwort einkreisen:   Ich mag Orangen.  0 1 2 3 4 5 6 7 8 9 10  überhaupt nicht sehr   - oder ein Kästchen ankreuzen:   An wie vielen Tagen pro Woche treiben Sie Sport?  0–1 Tag 🞎 2–3 Tage 🞎 4–5 Tage 🞎 6–7 Tage 🞎 |
| --- |

© Steven J. Linton, Örebro

| 1. In welchem Jahr sind Sie geboren? ............ |  |
| --- | --- |
| 2. Sind Sie männlich 🞎 weiblich 🞎 |  |
| 3. In welchem Land wurden Sie geboren? ........................... |  |
| 4. In welcher Beschäftigungssituation befinden Sie sich zurzeit?  bezahlte Arbeit 🞎 Studium 🞎 unbezahlte Arbeit zuhause 🞎  arbeitslos 🞎 pensioniert 🞎 andere 🞎 : |  |
| 5. Wo haben Sie Schmerzen? Es sind mehrere Antworten möglich.  Nacken 🞎 Schulter 🞎 oberer Rücken 🞎 unterer Rücken 🞎 Bein 🞎 | 2 × x |
| 6. An wie vielen Tagen konnten Sie, **während der letzten 12 Monate,** aufgrund von Schmerzen nicht Ihrer Arbeit nachgehen? Bitte wählen Sie eine Antwort.  0 Tage 🞎 1–2 Tage 🞎 3–7 Tage 🞎 8–14 Tage 🞎 15–30 Tage 🞎  31–60 Tage 🞎 61–90 Tage 🞎 91–180 Tage 🞎 181–365 Tage 🞎 > 365 Tage 🞎 |  |
| 7. Wie lange haben Sie dieses Schmerzproblem schon? Kreuzen Sie das entsprechende Kästchen an.  0–1 Woche 🞎 2–3 Wochen 🞎 4–5 Wochen 🞎 6–7 Wochen 🞎 8–9 Wochen 🞎  10–11 Wochen 🞎 12–23 Wochen 🞎 24–35 Wochen 🞎 36–52 Wochen 🞎 > 52 Wochen 🞎 |  |
| 8. Ist Ihre Arbeit körperlich anstrengend oder monoton? Kreisen Sie die zutreffende Antwort ein.  0 1 2 3 4 5 6 7 8 9 10  überhaupt nicht extrem  Ich arbeite nicht. 🞎 |  |
| 9. Wie **stark** waren Ihre Schmerzen innerhalb der **letzten Woche**? Kreisen Sie eine Antwort ein.  0 1 2 3 4 5 6 7 8 9 10  kein Schmerz schlimmster Schmerz |  |
| 10. Wie **stark**, auf einer Skala von 0 bis 10, war Ihr Schmerz **während der letzten drei Monate** im Durchschnitt? Kreisen Sie eine Antwort ein.  0 1 2 3 4 5 6 7 8 9 10  kein Schmerz schlimmster Schmerz |  |
| 11. Wie **oft** hatten Sie im Durchschnitt **während der letzten drei Monate** Schmerzen? Kreisen Sie eine Antwort ein.  0 1 2 3 4 5 6 7 8 9 10  nie immer |  |

| 12. Wie weit ist es Ihnen möglich den Schmerz, mit Dingen die Ihnen helfen, zu reduzieren? Kreisen Sie eine Antwort ein.  0 1 2 3 4 5 6 7 8 9 10  Ich kann ihn überhaupt nicht lindern. Ich kann ihn völlig zum Verschwinden bringen. | 10 – x |
| --- | --- |
| 13. Wie angespannt oder unruhig fühlten Sie sich **in der letzten Woche**? Kreisen Sie eine Antwort ein.  0 1 2 3 4 5 6 7 8 9 10  absolut ruhig und entspannt so angespannt und unruhig wie nie zuvor |  |
| 14. Wie niedergeschlagen fühlten Sie sich **in der letzten Woche**? Kreisen Sie eine Antwort ein.  0 1 2 3 4 5 6 7 8 9 10  überhaupt nicht extrem |  |
| 15. Wie hoch denken Sie, ist das Risiko, dass Ihre Schmerzen bleibend sind? Kreisen Sie eine Antwort ein.  0 1 2 3 4 5 6 7 8 9 10  kein Risiko hohes Risiko |  |
| 16. Wie schätzen Sie die Wahrscheinlichkeit ein, dass Sie **in sechs Monaten** wieder arbeiten können? Kreisen Sie eine Antwort ein.  0 1 2 3 4 5 6 7 8 9 10  nicht vorhanden sehr hoch |  |
| 17. Wie zufrieden sind Sie mit Ihrer Arbeitsstelle in Bezug auf Arbeitsabläufe, Vorgesetzte, Lohn, Aufstiegsmöglichkeiten, Arbeitskolleginnen und ‑kollegen?  0 1 2 3 4 5 6 7 8 9 10  überhaupt nicht zufrieden völlig zufrieden  Ich arbeite nicht. 🞎 |  |
| *Es folgen einige Aussagen von Patienten, die über ihre Schmerzen Auskunft gaben. Bitte geben Sie bei jeder Aussage an, welche Auswirkungen körperliche Aktivitäten wie Bücken, Lasten heben, Gehen oder Autofahren auf* ***Ihren*** *Rücken haben oder hätten.* |  |
| 18. Körperliche Aktivitäten verstärken meinen Schmerz.  0 1 2 3 4 5 6 7 8 9 10  überhaupt nicht einverstanden völlig einverstanden |  |
| 19. Wenn der Schmerz zunimmt, ist dies ein Zeichen, dass ich mit dem, was ich gerade tue, aufhören sollte, bis dieser wieder abgenommen hat.  0 1 2 3 4 5 6 7 8 9 10  überhaupt nicht einverstanden völlig einverstanden |  |

| 20. Mit meinem momentanen Schmerz sollte ich weder meine normalen Aktivitäten ausüben noch arbeiten.  0 1 2 3 4 5 6 7 8 9 10  überhaupt nicht einverstanden völlig einverstanden |  |
| --- | --- |
| *Es folgen Fragen zu fünf Aktivitäten. Kreisen Sie jeweils die Zahl ein, die Ihre momentane Möglichkeit, die entsprechende Aktivität auszuüben, am besten beschreibt.* |  |
| 21. Ich kann eine Stunde lang leichte Arbeiten verrichten.  0 1 2 3 4 5 6 7 8 9 10  Das ist mir wegen des Das kann ich, ohne dass der Schmerzes nicht möglich. Schmerz ein Problem darstellt. | 10 – x |
| 22. Ich kann eine Stunde lang gehen.  0 1 2 3 4 5 6 7 8 9 10  Das ist mir wegen des Das kann ich, ohne dass der Schmerzes nicht möglich. Schmerz ein Problem darstellt. | 10 – x |
| 23. Ich kann alltägliche Haushaltsarbeiten verrichten.  0 1 2 3 4 5 6 7 8 9 10  Das ist mir wegen des Das kann ich, ohne dass der Schmerzes nicht möglich. Schmerz ein Problem darstellt. | 10 – x |
| 24. Ich kann meine Wocheneinkäufe erledigen.  0 1 2 3 4 5 6 7 8 9 10  Das ist mir wegen des Das kann ich, ohne dass der Schmerzes nicht möglich. Schmerz ein Problem darstellt. | 10 – x |
| 25. Ich kann nachts schlafen.  0 1 2 3 4 5 6 7 8 9 10  Das ist mir wegen des Das kann ich, ohne dass der Schmerzes nicht möglich. Schmerz ein Problem darstellt. | 10 – x |

**BESTEN DANK FÜR IHRE UNTERSTÜTZUNG!**
